# Whole genome sequencing of a novel carrageenan-degrading bacterium *Photobacterium rosenbergii* and oligosaccharide preparation

**DOI:** 10.3389/fmicb.2025.1519074

**Published:** 2025-01-23

**Authors:** Jing Chen, Runmin Chen, Kit-Leong Cheong, Zhuo Wang, Rui Li, Xuejing Jia, Qiaoli Zhao, Xiaofei Liu, Bingbing Song, Saiyi Zhong

**Affiliations:** ^1^Shenzhen Research Institute, Guangdong Ocean University, Shenzhen, China; ^2^Guangdong Provincial Key Laboratory of Aquatic Product Processing and Safety, Guangdong Province Engineering Laboratory for Marine Biological Products, Guangdong Provincial Engineering Technology Research Center of Seafood, College of Food Science and Technology, Guangdong Ocean University, Zhanjiang, China

**Keywords:** *Photobacterium rosenbergii*, carrageenan, oligosaccharides, genome sequencing, preparation

## Abstract

**Introduction:**

Carrageenan oligosaccharides are of significant interest due to their diverse bioactivities, necessitating efficient methods for their production. To this day, the discovery and isolation of microorganisms capable of effectively degrading carrageenan is still crucial for the production of carrageenan oligosaccharides. In addition, there are no current reports of bacteria of the genus Photobacterium capable of secreting κ-carrageenanase or degrading carrageenan.

**Methods:**

In the current study, strain GDSX-4 was obtained from Gracilaria coronopifolia after enrichment culture, primary screening and rescreening and was initially characterized by morphology and 16SrDNA. The pure culture of strain GDSX-4 was further subjected to bacterial genome sequencing assembly and bioinformatic analysis. Specifically, homology group cluster (COG) annotation, CAZy (carbohydrate-active enzyme) database annotation and CAZyme genome clusters (CGCs) annotation were utilized to identify potential polysaccharide degradation functions. Enzymatic activity was assessed under different conditions, including substrate, temperature, pH, and the presence of metal ions. Hydrolysis products were analyzed using thin-layer chromatography (TLC) and electrospray ionization mass spectrometry (ESI-MS).

**Results:**

*Photobacterium rosenbergii* GDSX-4 is a Gram-negative bacterium isolated from the red algae, capable of degrading several polysaccharides. The draft genome was predicted to have 6,407,375 bp, 47.55% G+C content and 6,749 genes. Among them, 214 genes encoding carbohydrate enzymes were annotated, including carrageenase, agarose, alginate lyase, and chitinase. GDSX-4 exhibited remarkable carrageenan-degrading activity, with a specific enzyme activity of 46.94 U/mg. Optimal hydrolysis conditions were determined to be 40°C and pH 7.0, with the enzyme retaining 80% of its activity below 30°C and across a pH range of 4.0–10.0. Metal ions such as as K^+^, Na^+^, and Ba2^+^ enhanced enzymatic activity, while Ni2^+^, Mn2^+^, and Cu2^+^ had inhibitory effects. kappa-carrageenan was totally hydrolyzed into oligosaccharides with degrees of polymerization ranging from 2 to 6.

**Discussion:**

These findings highlight the potential of GDSX-4 for the efficient production of carrageenan oligosaccharides, paving the way for applications in the food and agricultural industries. Future studies may focus on the efficient expression of κ-carrageenase and expand its industrial application in the preparation of oligosaccharides.

## 1 Introduction

Carrageenan is a macromolecular linear sulfated polysaccharide extracted from red algae, such as *Gelidium*, *Chondrus*, and *Silvetia* (Gurpilhares et al., 2016), and processed into an off-white or light yellow to brownish-yellow powder or granule using water or alkaline solutions. The structure of carrageenan is primarily composed of disaccharide repeating units of α (1,4)-linked D-galactose (D) or 3,6-anhydro-D-galactose residue (DA) and β (1,3)-linked D-galactose residue (G) (Wallace et al., 2020). The three main types of carrageenan, κ-, ι-, and λ- carrageenan, are widely used in various industries, mainly in food, cosmetics, and pharmaceuticals (Liu et al., 2021). However, its high molecular weight and low bioavailability limit some application scenarios of carrageenan. Carrageenan oligosaccharides, which are degradation products of carrageenan and exhibit advantages such as low molecular weight and high solubility over the original polysaccharides (Guo et al., 2022). Additionally, due to the sufficient exposure of the reactive groups, the bioactivity is significantly improved compared with that before degradation (Humayun et al., 2024).

In general, the main methods for obtaining carrageenan oligosaccharides include chemical, physical, and biological approaches. Carrageenan oligosaccharides prepared using these different methods exhibit significant variations in their structure, content of active functional groups, degree of polymerization distribution, and batch-to-batch reproducibility (Zheng J. et al., 2023). The traditional chemical degradation method for the preparation of oligosaccharides presents several challenges, such as harsh reaction conditions that are difficult to control, complex product analysis and purification processes, difficulties in recycling chemical reagents, and significant environmental pollution (Guo et al., 2022). In contrast, the biodegradation method employs carrageenase to degrade polysaccharides to prepare carrageenan oligosaccharides with varying degrees of polymerization (Ghanbarzadeh et al., 2018). This process occurs under mild reaction conditions, with high catalytic efficiency, no pollution, and good substrate specificity. Furthermore, it allows for the selective cutting of glycosidic bonds on the carrageenan glycan chain, it offers a sustainable and eco-friendly approach (Guo et al., 2022).

Currently, a variety of genera with κ-carrageenase secretion capacity have been identified from the marine environment, such as *Pseudoalteromonas* (Lemoine et al., 2009; Kobayashi et al., 2014), *Pseudomonas* (Khambhaty et al., 2007), *Shewanella* (Wang et al., 2015), *Vibrio* (Liu et al., 2016), *Flammeovirga* (Gao et al., 2017), *Cellulophaga* (Yao et al., 2013), *Cytophaga* (Haijin Mou et al., 2004), *Tamlana* (Sun et al., 2010), *Polaribacter* (Gui et al., 2021), *Pedobacter* (Sun et al., 2014), *Zobellia* (Thomas et al., 2017), *Rhodopirellula* (Zhang et al., 2022). These bacteria exhibit diverse enzymatic properties, but despite this variety, no κ-carrageenase has yet been applied on an industrial production scales (Zhu et al., 2018b; Xiao et al., 2018). Consequently, the continued discovery and isolation of microorganisms capable of efficiently degrading carrageenan remains essential for the production of carrageenan oligosaccharides. Notably, to date, there have been no reports of bacteria from the genus *Photobacterium* being able to secrete κ-carrageenase or degrade carrageenan.

In the present study, *Photobacterium rosenbergii* GDSX-4 was isolated and identified from red algae (*Gracilaria coronopifolia*). Using whole-genome sequencing technology, the GDSX-4 strain was further identified, and the genetic basis of its polysaccharide degradation capability was explored. The isolate strain’s capacity to degrade various polysaccharides, ideal enzymatic conditions, stability, and the enzymatic products were investigated. GDSX-4 contains abundant enzymes and the ability to degrade several polysaccharides, with the strongest ability to degrade κ-carrageenan. GDSX-4 may be a source of κ-carrageenase, which can be used for the preparation of carrageenan oligosaccharides and potential biotechnological applications.

## 2 Materials and methods

### 2.1 Materials

*Gracilaria coronopifolia* was obtained from Prof. Enyi Xie’s laboratory (Guangdong Ocean University, Zhanjiang). Kappa-carrageenan, lambda-carrageenan and iota-carrageenan were purchased from Sigma-Aldrich. Kappa-neocarrabiose sulfate sodium salt, kappa-neocarratetraose disulfate disodium salt, and kappa-neocarrahexaose trisulfate trisodium salt were procured from Qingdao HEHAI Biotech Co., Ltd. (Qingdao, China). Chemicals such as peptone, diphenylamine, calcium chloride dehydrates, sodium alginate, phosphoric acid, potassium hydrogen phosphate, and sodium chloride were procured from Xilong Chemical Co., Ltd. (China). Lugol’s iodine solution and dinitro salicylic acid were obtained Beijing Solarbio Science & Technology Co., Ltd. Other reagents used in the present study were of analytical grade.

### 2.2 Isolation and identification of *Photobacterium rosenbergii* GDSX-4

*Gracilaria coronopifolia* was crushed and cultivated in enrichment medium, purified repeatedly streaking onto solid plate until a single colony was obtained. The solid plate containing 15 g/L carrageenan, 5 g/L peptone and 1 g/L yeast extract dissolved in 1 L filtered sterilized old seawater. Following that, bacteria were incubated on medium with carrageenan as the sole carbon source at 30°C for 48 h, stained with 5 mL of Lugol’s iodine solution and the hydrolysis circle was detected to evaluate the ability to produce carrageenan lyase. The screened strains were subjected to Gram staining and the morphology of the strains was observed by microscopy. Biochemical identification using Gram-negative bacterial identification system (Qingdao Hi-Tech Industrial Park Hope Bio-Technology Co., Ltd.). DNA of single colonies was extracted, and the 16S rRNA gene sequence was amplified using universal primers 27F and 1492R. DNA was extracted from single-colony cultures, and the full-length 16S rRNA gene was amplified using the universal primers 27F (5′-AGTTTGATCMTGGCTCAG-3′) and 1492R (5′-GGTTACCTTGTTACGACTT-3′). The PCR reaction system was prepared in a total volume of 25 μL, consisting of 1 μL of DNA template, 1 μL of each primer, 2.5 μL of 10× PCR Buffer, 0.5 μL of dNTP Mix (10 mM), 0.2 μL of Taq polymerase (5 U), and nuclease-free water to make up the final volume. The amplification conditions were pre-denaturation 95°C hold for 5 min, 30 s at 94°C, 30 s at 57°C, 90 s at 72°C, 30 cycles, and 10 min at 72°C. The PCR products were then purified and subjected to DNA sequencing for further analysis. The sequencing results were submitted to the EZbio database and NCBI database for comparison, and the phylogenetic tree of the strain GDSX-4 was constructed by multiple sequence comparison using MEGA11.

### 2.3 Genome sequencing and assembly

*Photobacterium rosenbergii* GDSX-4 was cultured in nutrient medium at 30°C for 48 h. Then, cells were collected at 4,000 × g for 10 min through centrifugation. Genomic DNA was extracted with the SDS method (Lim et al., 2016). The purity of genomic DNA was detected by 1% agarose gel electrophoresis and quantified by Qubit Fluorometer (Thermo Scientific). Library construction and the whole genome of *Photobacterium rosenbergii* GDSX-4 was sequenced using PacBio Sequel platform and Illumina NovaSeq PE150 at the Beijing Novogene Bioinformatics Technology Co., Ltd. After passing the library inspection, Illumina PE150 was used to obtain raw data. Then the sequencing data were filtered and processed, and the obtained Clean Data was used for subsequent analysis. Based on the above sequencing data, genome assembly of reads was performed using Canu software.^1^

### 2.4 Gene function

Coding DNA sequences (CDSs) were predicted using GeneMarkS (Version 4.17).^2^ Based on sequence composition, IslandPath-DIOMB software (Version 0.2) was used to predict gene islands. Gene annotation and functional prediction was accomplished using GO (Gene Ontology), KEGG (Kyoto Encyclopedia of Genes and Genomes), and COG (Cluster of Orthologous Groups of proteins). Use Diamond software to compare the amino acid sequences of the target species with the CAZy (Carbohydrate-Active Enzymes) database,^3^ and combine the genes of the target species with their corresponding functional annotations to obtain the annotation results. CAZyme gene clusters (CGCs) and substrate prediction by annotated dbCAN3 server (unl.edu) (Zheng J. et al., 2023).

### 2.5 Polysaccharide degradation capacity

The degradation ability was performed using the DNS method to detect the increase in reducing sugar content in the reaction system (Zhu et al., 2018a). The reaction mixture consisted of 50 μL of bacterial suspension, 450 μL of 0.2% polysaccharide substrate (κ-, ι-, or λ-carrageenan, agar, alginate) in phosphate buffer (pH 8.0), and was incubated at 40°C for 30 min. After adding 500 μL of DNS reagent to the reaction and thoroughly mixing it, the reaction was allowed to boil for 10 min to develop the color. The absorbance value was then measured at OD_540_, and galactose was used to create the standard curve. An equal amount of inactivation reaction solution served as the control. A unit of enzyme activity (U) is defined as the amount of enzyme required to catalyze the production of 1 μg of reducing sugar (in terms of D-galactose) per min under the current conditions.

### 2.6 Effect of metal ions on GDSX-4 activity

To investigate the effect of different metal ions on the degradation activity, metal ion reagents of NiCl_2_, CuCl_2_, FeSO_4_, MnSO_4_, MgSO_4_, ZnSO_4_, CaCl_2_, KCl, BaCl_2_, and NaCl were added to the reaction system at a final concentration of 5 mmol/L, respectively, and the reaction was carried out without metal ions as a control, and the degradation capacity was determined by reacting the reaction at 40°C for 30 min.

### 2.7 Optimum reaction temperature and thermal stability of GDSX-4

The bacterial suspensions were reacted at 0–70°C for 30 min to determine the degradation ability, respectively, and the degradation capacity at the optimum reaction temperature was taken as 100%. Meanwhile, equal amounts of bacterial solutions were treated at 30, 40, 50, and 70°C, and samples were taken at 1 h intervals to measure residual activity at the optimum reaction temperature.

### 2.8 Optimal reaction pH and pH stability for GDSX-4

The 0.2% carrageenan substrate was prepared with buffers of different pH, and the degradation capacity was determined at the optimum reaction temperature, with the highest activity as 100%. Reactions were carried out at pH 3.0–5.0 (C_6_H_8_O_7_-Na_2_HPO_4_), pH 6.0–8.0 (Na_2_HPO_4_-NaH_2_PO_4_), pH 9.0–11.0 (Na_2_CO_3_-NaHCO_3_) at the optimum reaction temperature. The pH stability was determined by mixing the bacterial solution with equal amounts of different pH (3.0–11.0) buffers and leaving it at 4°C for 24 h. The residual activity was determined by taking the degradation activity when untreated as 100%.

### 2.9 Hydrolysis of products analysis by thin layer chromatography

The bacterial solutions were mixed with 0.5% κ-carrageenan substrate and reacted at 40°C for 72 h. Samples were taken at 10, 15, 30, 45 min, 1, 2, 3, 6, 12, 24, 27, 30, 48, 72 h for TLC analysis. All the above-mentioned reacted carrageenan oligosaccharide and oligosaccharide standards were analyzed on TLC Silica gel 60 plates from Merck Germany (10 × 20 cm). The products of different degrees of polymerization were separated in the mobile phase of n-Butanol, isopropanol, acetic acid and water in the ratio 7:5:2:1. The TLC plate loaded with 3 μL of reactants was kept in the mobile phase for 60 min, sprayed with aniline-diphenylamine-phosphoric acid as the color developer, and dried at 130°C for 5 min until distinct spots appeared.

### 2.10 ESI-MS analysis of degradation products of GDSX-4

To determine the degree of polymerization of the hydrolysis products, the above hydrolyzed products were diluted to 50 μg/mL, filtered through 0.22 μm microporous membrane and injected 1 μL into the system for analysis. Chromatographic analysis was performed on a Waters UPLC system equipped with an acquity UPLC® BEH C_18_ column (1.7 μm, 2.1 × 50 mm) at 35°C. The gradient elution was performed using 0.1% formic acid aqueous solution (A) and acetonitrile solution (B) at a flow rate of 0.2 mL/min, and the gradient program was as follows: 0 min 2% B, 0–7 min 2–50% B, 7–8 min 50% B, 8–12 min 98% B. Mass spectrometry was performed on a Waters Xevo G2-XS Qtof mass spectrometer equipped with an ESI source. The mass spectrometry source conditions included: source and desorption gas temperatures of 120°C and 450°C, respectively, a desorption gas (N_2_) flow rate of 700 L/h, and a sampling cone voltage of 40 V and a capillary voltage of 1.8 kV in the negative ion mode. The detection was carried out in a full-scan mode with a mass range of 50–2,000 Da.

## 3 Results

### 3.1 Separation and identification of *Photobacterium rosenbergii* GDSX-4

**Figure 1:**
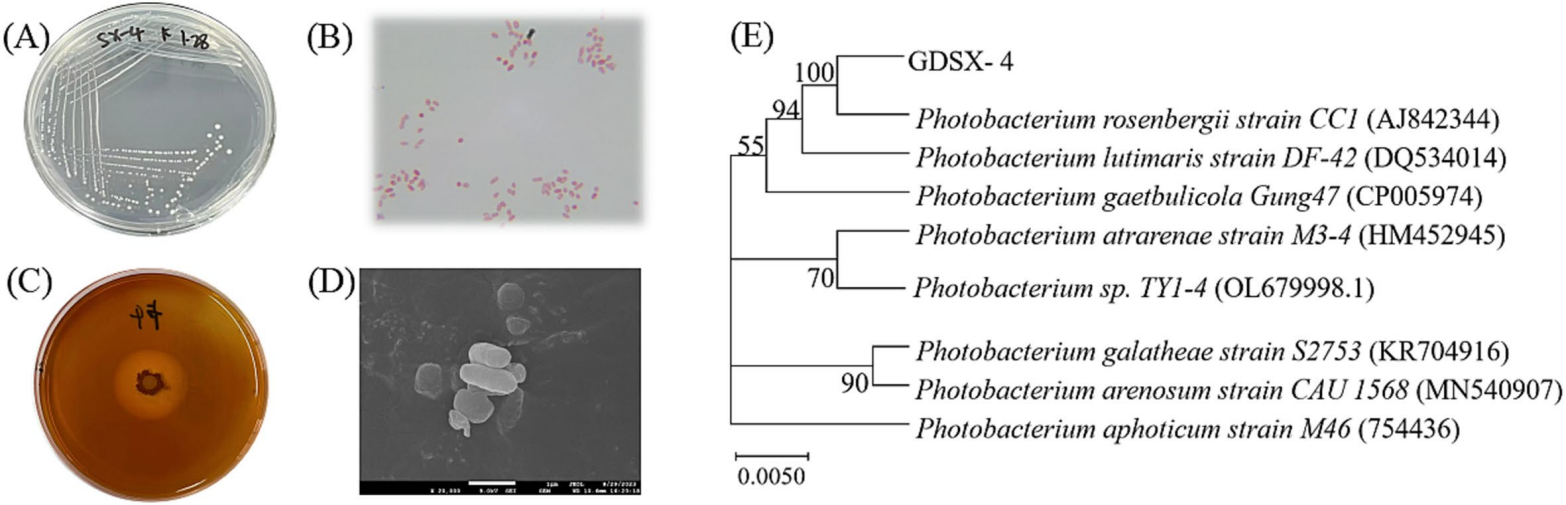
Characterization of GDSX-4 **(A)** Colony morphology **(B)** Gram strain **(C)** Iodine dye hydrolyzed rings **(D)** SEM image **(E)** Phylogenetic tree of GDSX-4 based on 16SrDNA sequences and Neighbor Joining analysis.

The strain GDSX-4 was successfully isolated from the red algae *Gracilaria coronopifolia*, the strain grew well in solid screening medium containing carrageenan (Figure 1A). The single colony was round, with neat edges, moist and glossy surface, creamy white, moderate size and easy to pick up, and Gram-negative stain (Figure 1B). Stained with Lugol’s iodine solution, the transparent hydrolysis circle of the strain to carrageenan could be clearly seen, and the diameter of the hydrolysis circle was about 3.0 cm (Figure 1C). The cells of the strain were short rod-shaped, with a length of 0.5–2 μm and a width of 0.21–0.5 μm (Figure 1D). The bacterial 16S rDNA sequence comparison showed 99.93% homology with *Photobacterium rosenbergii*, and the phylogenetic tree was constructed using Neighbor Joining as shown in Figure 1E, and strain GDSX-4 belonged to the genus *Photobacterium*. The biochemical features results of GDSX-4 as shown in Table 1, the strain was positive for β-galactosidase, indole, oxidase, glucose, D-mannitol, sucrose and melibiose, it was negative for others. *Photobacterium rosenbergii* was first isolated from coral by Thompson (Thompson et al., 2005), and the biochemistry of the bacterium is consistent with its reported ability to utilize glucose, D-mannitol, sucrose and melibiose.

**Table 1:** Biochemical identification results of GDSX-4.

Characteristics	Result	Characteristics	Result
β-galactosidase	+	Gelatin	**−**
Arginine dihydrolase	−	Glucose	**+**
Lysine dihydrolase	−	D-Mannitol	**+**
Ornithine decarboxylase	−	Inositol	**−**
Citrate	−	D-Sorbitol	**−**
H_2_S production	−	Rhamnose	**−**
Urease	−	Sucrose	**+**
Lactose	−	Melibiose	**+**
Indole	+	D-amygdalin	**−**
Voges-Proskauer	−	Arabinose	**−**
Oxidase	+		
NaCl tolerance Optimal	1–5 (3)	Range of temperature Optimal (°C)	10–42 (37)

“+” means positive; “−” means negative.

### 3.2 General features of *Photobacterium rosenbergii* GDSX-4 genome

**Figure 2:**
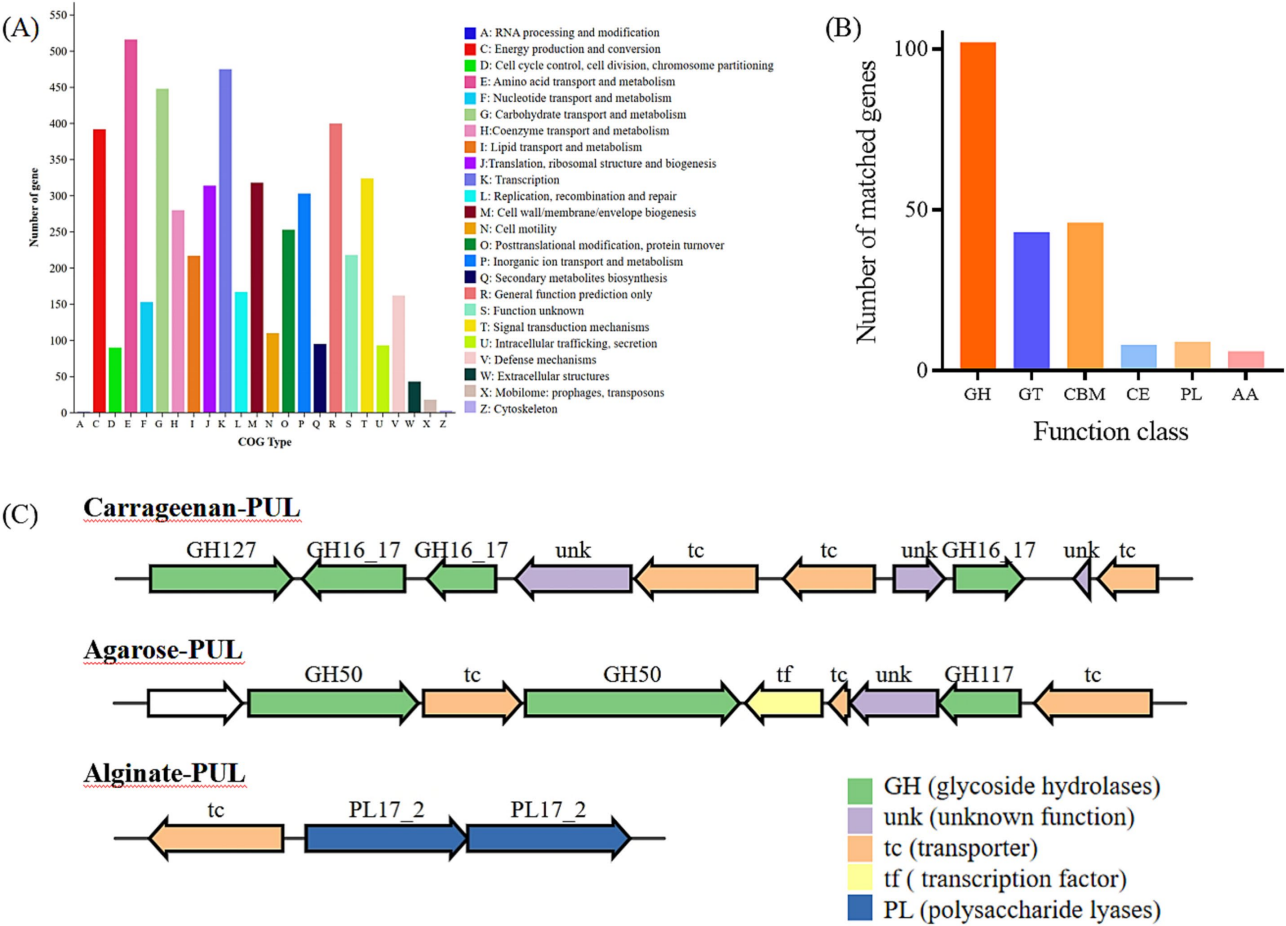
Annotation of COG functional classification **(A)**, Annotation of carbohydrate-related genes in the CAZy database **(B)**. GH, glycoside hydrolases; GT, glycosyl transferases; CBM, carbohydrate binding modules; CE, carbohydrate esterases; PL, polysaccharide lyases; AA, auxiliary activities. **(C)** PULs predicted Cluster of gene associated with carrageenan, agarose and aliginate degradation of *Photobacterium rosenbergii* GDSX-4.

The whole genome of strain GDSX-4 contained two circular chromosomes spanning 2,433,509 bp with 46.23%GC, and 3,973,866 bp with 48.68%GC, respectively (Table 2). Thompson has also reported that the G + C content of the DNA of the *Photobacterium rosenbergii* sp. nov. are 47.6 mol% (Thompson et al., 2005). According to the annotation results from the Non-Redundant Protein Database, we found that 2,666 genes had a high degree of protein sequence similarity with the *Photobacterium rosenbergii* with high protein sequence similarity, while 2,303 genes were annotated to the genus *Photobacterium*. In addition, the average nucleotide identity (ANI) scores between GDSX-4 and its closely related species were calculated as shown in Table 3, and the ANI values between GDSX-4 and *Photobacterium rosenbergii* DSM 19138, *Photobacterium rosenbergii* DP6N14–7 had ANI values greater than 95%. While the lowest ANI score with *Photobacterium rosenbergii* HSC6 was 87.02%, which may be due to its genome being a bit smaller than GDSX-4. Therefore, these data strongly suggest that strain GDSX-4 belongs to the species *Photobacterium rosenbergii* and was named as *Photobacterium rosenbergii* GDSX-4. The total length of coding genes was 6,058,089 bp, 499 interspersed nuclear elements, 301 tandem repeats finder, 215 tRNA, the number of 5 s rRNA, 16s rRNA and 23s rRNA was 23, 22, and 22, respectively. In addition, functional annotations were made for 2,971, 5,984, 5,984, 4,797, and 4,544 proteins from Swiss-Prot, KEGG, COG, GO databases, respectively (Table 2). As shown in Figure 2A, the 964 genes in the COG annotation results were related to amino-acid and carbohydrate transport and metabolism, which predicted that GDSX-4 has a strong potential for carbohydrate and amino acid metabolism. The GO annotation results focused on genes related to polysaccharide degradation process, 135 genes were annotated as carbohydrate metabolic process (GO:0005975), 228 genes with hydrolase activity (GO:0016787), 49 genes with lyase activity (GO:0016829), and 53 genes annotated as hydrolase O-glycosyl activity (GO:0004553). There were 5,984 genes functionally annotated in the KEGG database, of which 387 genes were related to carbohydrate metabolism, and two kappa-carrageenase genes were annotated as gene478, gene483 (K20846), suggesting that these genes play a key role in the degradation and utilization of carrageenan by GDSX-4.

**Table 2:** General features of the *Photobacterium rosenbergii* GDSX-4.

Properties	Value
Size	6.41 mbp
GC%	47.55
Completeness %	99.25
Contamination%	9.10
N50(bp)	857,947
Gene number	6,749
Gene average length	898
Gene length/genome	84.41
tRNA	215
rRNA	67
GIs number	8
NR	6,344
Swiss-Prot	2,971
Pfam	4,544
COG	4,797
GO	4,544
KEGG	5,984

**Table 3:** ANI score of *Photobacterium rosenbergii* GDSX-4 with its closely related strains.

	*Photobacterium rosenbergii* DSM 19138	*Photobacterium rosenbergii* HSC6	*Photobacterium rosenbergii* DP6N14–7	*Photobacterium gaetbulicola* DSM26887	*Photobacterium gaetbulicola* AD005a	*Photobacterium gaetbulicola* Gung 47	*Photobacterium lutimaris* CECT 7642	*Photobacterium atrarenae* GJH2-4	*Photobacterium galatheae* DSM 100496
**Average nucleotide identity**									
OrthoANIu value (%)	95.76	87.02	97.02	82.99	81.50	83.33	81.50	75.02	72.72
Genome A length (bp)	6,407,375	6,407,375	6,407,375	6,407,375	6,407,375	6,407,375	6,407,375	6,407,375	6,407,375
Genome B length (bp)	6,316,860	5,917,020	6,394,380	5,834,400	5,925,180	5,908,860	5,925,180	5,404,980	5,447,820
Average aligned length (bp)	3,938,540	3,192,582	4,279,778	3,073,418	2,793,055	2,958,622	2,793,055	1,647,366	1,363,647
Genome A coverage (%)	61.48	49.83	66.80	47.97	43.60	46.18	43.60	25.71	21.28
Genome B coverage (%)	62.35	53.96	66.93	52.68	47.14	50.07	47.14	30.48	25.03

### 3.3 Predicted carbohydrate-active enzyme in *Photobacterium rosenbergii* GDSX-4

To search for genes linked to polysaccharide related degrading enzymes in the genome of *Photobacterium rosenbergii* GDSX-4, the carbohydrate-active enzymes were annotated using the CAZy database, a total of 214 carbohydrate-related genes were identified (Figure 2B). Among these, 102 were glycoside hydrolases (GH), followed by 46 carbohydrate binding modules (CBM), and 43, 8, 9, and 6 for glycosyl transferases (GT), carbohydrate esterases (CE), polysaccharide lyases (Hettle et al.), and auxiliary activities (AA), respectively. These results anticipated the presence of the GH16_17 family, which has been classified as κ-carrageenase (EC 3.2.1.83) (Zhu et al., 2018b), the major products of hydrolysis being neocarrabiose-sulfate and neocarratetraose-sulfate. Additionally, the GH127 family, which also hydrolyzes carrageenan, produces α-1,3-(3,6)-anhydro-D-galactose (Ficko-Blean et al., 2017). Another predicted GH16_21 family known as beta-glucanase (EC 3.2.1.73) (Viborg et al., 2019), hydrolysis of β (1,4)-glucosidic linkages in beta-glucans containing (1,3)- and (1,4)- bond. Two β-agarase genes belonging to GH50 were identified, showing high amino-acid similarity (80.1 and 88.2%) to β-agarase from *Photobacterium gaetbulicola* (AJR05318.1), and the main degradation product of neoagarotetraose (Jiang et al., 2022). As shown in Table 4, PL families such as PL6, PL7 and PL17_2 were predicted, which are involved in alginate degradation (Ji et al., 2017).

**Table 4:** Polysaccharide-degrade enzymes in *Photobacterium rosenbergii* GDSX-4 genome.

GH Family (Cazy)	% of genes	GH Family (Cazy)	% of genes	GH Family (Cazy)	% of genes
CBM32	2.010050	CE0	0.502513	GH35	0.502513
CBM48	0.502513	CE11	0.502513	GH36	1.507538
CBM5	0.502513	CE4	1.507538	GH38	1.507538
CBM50	11.557789	CE8	0.502513	GH4	1.005025
CBM56	0.502513	CE9	1.005025	GH43_3	0.502513
CBM6	0.502513	GH149	0.502513	GH50	1.005025
CBM73	0.502513	GH166	0.502513	GH5_18	0.502513
GH0	1.507538	GH16_17	1.507538	GH6	0.502513
GH1	3.517588	GH16_21	1.005025	GH63	0.502513
GH100	1.005025	GH170	1.005025	GH65	0.502513
GH102	0.502513	GH171	0.502513	GH73	1.005025
GH103	0.502513	GH18	1.507538	GH76	0.502513
GH114	0.502513	GH2	1.507538	GH77	0.502513
GH117	0.502513	GH20	1.507538	GH9	1.005025
GH127	0.502513	GH23	4.522613	GH94	1.005025
GH13	8.542713	GH26	0.502513	GT0	1.005025
GT5	1.005025	GH28	0.502513	GT1	2.010050
GT51	3.015075	GH3	4.020101	GT100	0.502513
GT83	0.502513	GH32	3.015075	GT19	1.005025
GT87	1.005025	PL15	1.005025	GT28	1.507538
GT9	1.005025	PL22	0.502513	GT30	1.005025
GT99	0.502513	PL38	0.502513	GT35	0.502513
PL17_1	0.502513	PL6	0.502513	GT4	7.035176
PL17_2	1.005025	PL7	0.502513	AAs	2.336448

The family highlighted in Red are indicating the % of genome coding for carrageenase belonging to GH16_17 and GH127 family. The GH50, GH117, PL6, PL7and PL17_2 families highlighted in Green are indicating the % of genome coding for agarose and alginate lyase.

The polysaccharide utilization sites (PULs) encoded in the *Photobacterium rosenbergii* GDSX-4 genome were further investigated, including those for carrageenan, agar, and alginate, are shown in Figure 2C. The carrageenan polysaccharide utilization locus contained 10 consecutive genes (Figure 2C) with complete structure, containing one gene coding for GH127 at the start end, three other genes coding for GH16_17, three transporter proteins and three unknown functional proteins. Two of these putative GH16_17 (GM000478, 000483) have 56.8 and 57.5% amino acid homology, respectively, with κ-carrageenase of *Pseudoalteromonas carrageenovora* origin (Guibet et al., 2007). κ-carrageenase of the GH16 family cleaves the polysaccharide chains into smaller oligosaccharide chains, and GH127 hydrolyzes carrageenan disaccharide to 3,6-anhydro-D-galactose and galactose (Thomas et al., 2017). The agar polysaccharide utilization locus contains nine contiguous genes (Figure 2C), four transporter proteins, one transcription factor and one protein of unknown function, with two genes encoding GH50 β-agarases (GM001361, 001363) and one gene encoding GH117 (GM001367). A query of the CAZy database indicates that the GH117 family contains β-D-galactofuranosidase (EC 3.2.1.146) and α-neoagaro-oligosaccharide hydrolase (EC 3.2.1.159) (Tsevelkhoroloo et al., 2023). Another gene cluster contained two genes coding for PL17-2 Alginate lyases and one transcription protein, which presumably can utilize alginate as a substrate. Thus, the prediction of polysaccharide-degrading glycolytic enzymes reported to carry different families of polysaccharides indicated that the GDSX-4 may have the potential to hydrolyze polysaccharides such as carrageenan, agar, and alginate.

### 3.4 Polysaccharide degradation ability of *Photobacterium rosenbergii* GDSX-4

**Figure 3:**
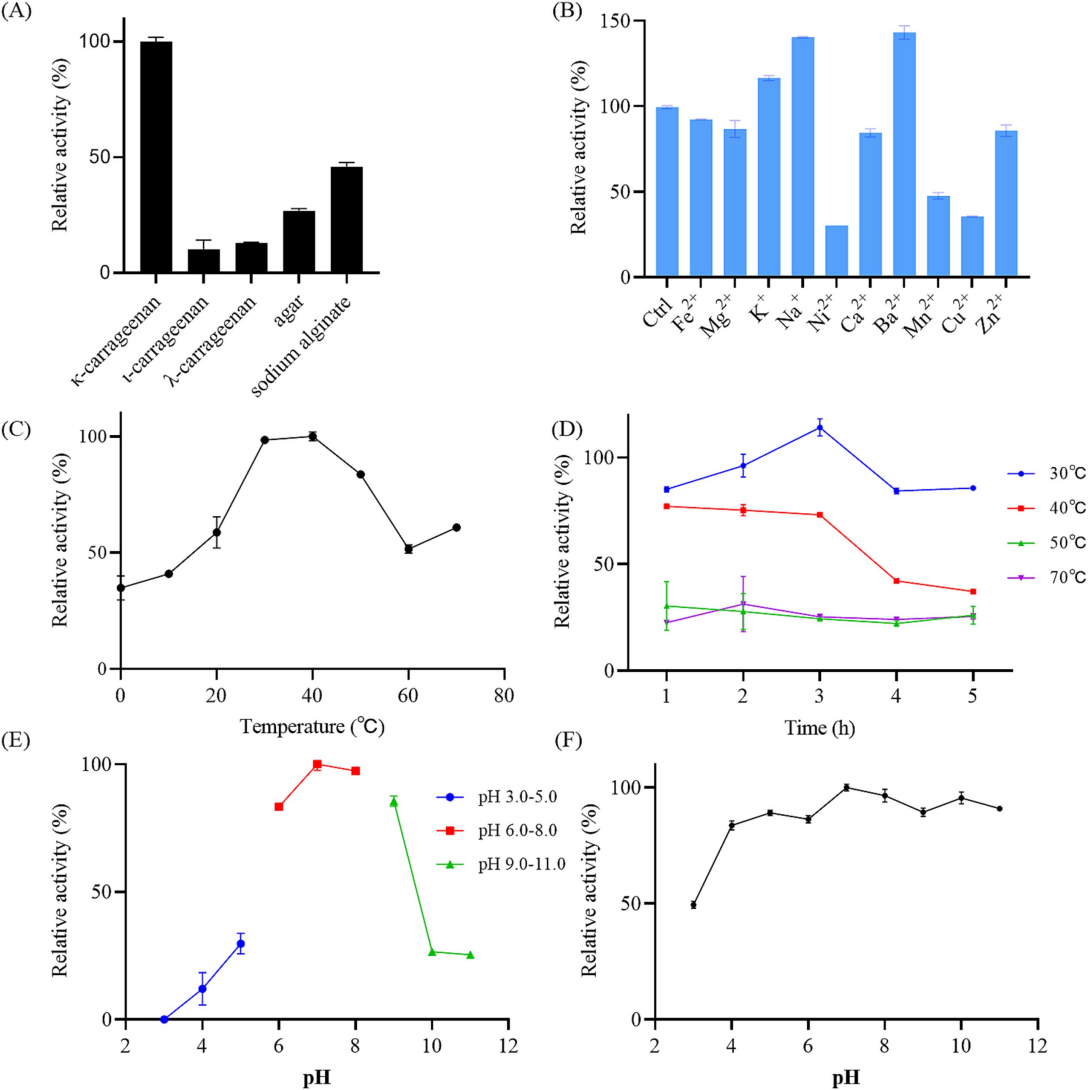
Characterization of reaction conditions of the GDSX-4. **(A)** The bacterial solutions reacted with different substrate. **(B)** Effects of the metal ions on relative activity. Ctrl presents control group without any other metal ion added. **(C)** Effects of temperature on the activity. **(D)** Thermal stability of the bacterial solutions by incubating at 30, 40, 50, and 70°C for 5 h. **(E)** Optimal pH. **(F)** The pH stability. Values given were expressed as mean ± SD, *n* = 3.

To further probe the polysaccharide degradation capacity of the GDSX-4, we incubated the bacterial solution with κ-carrageenan, λ-carrageenan, ι-carrageenan, agar and solidum alginate. As shown in Figure 3A, GDSX-4 exhibited notable enzymatic activity against the κ-carrageenan, followed by sodium alginate, and minor agar degradation, but not ι-carrageenan and λ-carrageenan. It is speculated that the degradation ability of different polysaccharides is related to the number of genes of hydrolase enzymes, and as shown in Table 4, the predicted genes for κ-carrageenan hydrolase are the most abundant, followed by alginate lyase genes. The presence of alginate lyase and agarase in the genus *Photobacterium* has also been reported by others, Lu et al. identified two novel alginate lyases from *Photobacterium* sp. FC615 (Lu et al., 2019), Jiang et al. cloned a novel GH50 β-agarose from *Photobacterium gaetbulicola* (Jiang et al., 2022), whereas there have been no reports on the degradation of carrageenan. Considering the GDSX-4 showed comprehensive κ-carrageenase activity and considered as a decomposition bacterium of κ-carrageenan. Therefore, in the subsequent tests, only κ-carrageenan was employed as a substrate, although GDSX-4 has the potential to degrade several substrates.

### 3.5 Optimal conditions for the preparation of oligosaccharides by GDSX-4

The optimal reaction conditions of GDSX-4 were further determined. The results revealed that the highest activity was observed at pH 7.0 and 40°C, indicating the GDSX-4’s preference for neutral pH and moderate temperatures (Figures 3C,E). The thermal stability results are shown in Figure 3D, indicating that GDSX-4 retained more than 85% activity after incubation at 30°C for 5 h, and also retained more than 70% of the activity after 3 h of incubation at 40°C. Moreover, Figure 3F shows that the GDSX-4 maintained 80% activity was stable over the pH range of 4.0–11.0. Most of the reported optimum temperatures and pH for carrageenase are 30–40°C (Zhu et al., 2018b) and 6.0–8.0 (Jiang et al., 2024), respectively, which are consistent with GDSX-4. The influence of various metal ions on GDSX-4 activity are shown in Figure 3B, Fe^2+^, Mg^2+^, Ca^2+^, and Zn^2+^ could slightly reduce the activity, while Ni^2+^, Mn^2+^, and Cu^2+^ could significantly inhibit the activity. Remarkably, K^+^, Na^+^, and Ba^2+^ significantly enhanced degraded activity, which increased it to 17, 40, and 43% of its initial activity, respectively. In most cases, Na^+^ and K^+^ can promote the play of enzyme activity (Zhu et al., 2018b), which is consistent with the results of this study.

### 3.6 Analysis of the hydrolysates

**Figure 4:**
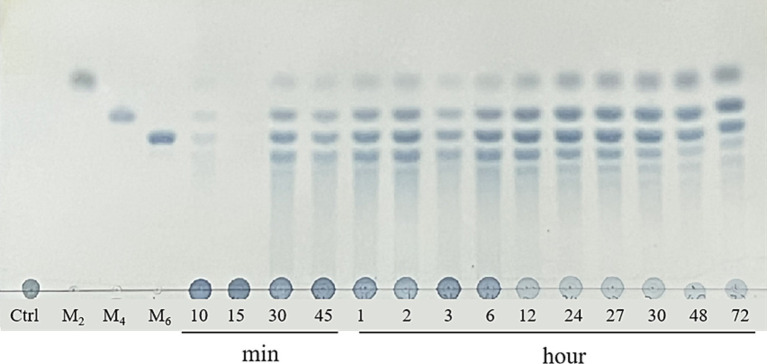
TLC analysis of the hydrolysis of kappa-carrageenan with GDSX-4 at different times. Lane M_2_, M_4_, M_6_ represent standards for disaccharides, tetrasaccharides and hexasaccharides.

**Figure 5:**
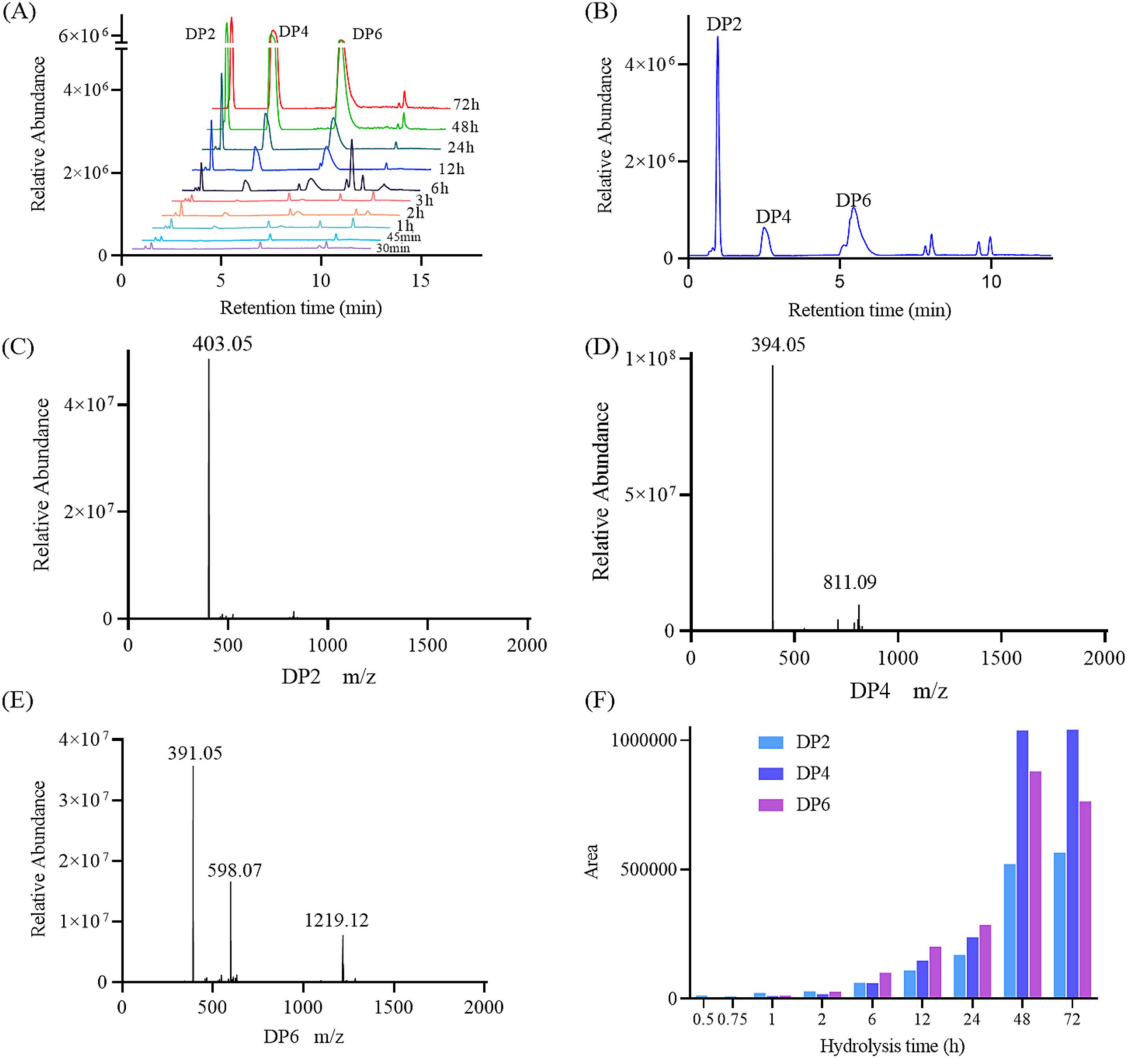
The ESI-MS analysis of hydrolysate. **(A)** TIC for hydrolyte products at different times. **(B)** Standards for disaccharides, tetrasaccharides and hexasaccharides. Mass spectrometry of DP2 **(C)**, DP4 **(D)**, DP6 **(E)**, Spectrum area **(F)**.

TLC was used to analyze the degradation products initially. As shown in Figure 4, only few oligosaccharides were generated in the system before 30 min of the GDSX-4 degradation reaction. Following this period, the system primarily produced κ-neocarrabiose, κ-neocarratetraose, and κ-neocarrahexaose, along with a few oligosaccharide fractions with aggregation degrees greater than six. The concentration of oligosaccharide spots increased as the degradation time was extended. Especially after 48 h of the reaction, the oligosaccharides with a degree of polymerization greater than six decreased dramatically, which might be that a portion of oligosaccharides was further degraded into κ-neocarrabiose. To ascertain the oligosaccharide composition even more, ESI-Q-TOF-MS was employed. In order to investigate the hydrolysis pattern of GDSX-4, the changes in oligosaccharide production between 0.5 and 72 h of GDSX-4 hydrolysis were detected as shown in Figure 5A. A distinct response peak was visible in the products before 30 min of the reaction, which were dominated by κ-neocarabinose. A minor amount of both κ-neocarratetraose and κ-neocarrahexaose were produced, but the response value was incredibly low and the peak area could not be integrated. The response peaks of the three oligosaccharide fractions were clearly visible at 1 h into the hydrolysis reaction, and as time went on, the generation of the three oligosaccharide fractions increased steadily. Particularly after 48 h, product generation peaked, and longer times did not result in the production of any more products.

The κ-neocarabinose, κ-neocarratetraose, and κ-neocarrahexaose mixed standards were subjected to chromatographic as shown in Figure 5B. In accordance the literature, the peaks at m/z 403, m/z 394 or 811, m/z 598 or 1,219 represent disaccharides [(An-G4S)]^−^, tetrasaccharides [(An-G4S)_2_]^2−^, hexasaccharides [(An-G4S)_3_]^−^, respectively (Wallace et al., 2020; Wei et al., 2023). As can be seen from Figure 5C, the ionic peaks at 403.05 corresponding to κneocarrabiose. As shown in Figure 5D, the peaks at m/z 394.05 ([(An-G4S)_2_]^2−^) and m/z 811.09 ([(G4SAn)_2_Na]^−^) were assigned to κ-neocarratetraose, and all the species at m/z 391.05([(An-G4S)_3_]^3−^), 598.07 ([(An-G4S)_3_Na]^2−^) and 1219.12([(An-G4S)_3_2Na]^−^) (Figure 5E) could be attributed to κ-neocarrahexaose. By using the peak area integral calculation, as seen in Figure 5F, all of the degradation products were produced at the end of the hydrolysis procedure. Of these, 64.34% were κ-neocaratetraose, 20.88% were κ-neocarabinose, and 14.78% were κ-neocarrahexaose. This result is consistent with the major degradation products of κ-carrageenan from *Pseudoalteromonas fuliginea* PS47 (Hettle et al., 2019).

## 4 Discussion

In this study, we successfully isolated a marine bacterial strain from the red alga, *Photobacterium rosenbergii* GDSX-4, which has the ability to efficiently degrade κ-carrageenan. The genome characterization and functional genes of the bacterium were revealed by whole-genome sequencing, which revealed a large number of genes of carbohydrate-active enzymes and polysaccharide utilization loci, such as carrageenase, agarose and alginate lyase, which is closely related to its strong polysaccharide degradation ability. Similarly, whole-genome sequencing has identified polysaccharide-degrading genes in various bacterial genera. Li reported that a gene sketch of the *Pseudoalteromonas* sp. strain (Li et al., 2017) predicts multiple polysaccharide degrading genes, such as agar, alginate and chitin. Gao identified a large number of enzyme genes involved in polysaccharide degradation in *Flammeovirga pacifica* strain WPAGA1 (Gao et al., 2017), including cellulase, amylase, alginate lyase, chitinase, carrageenase, fucosidase. Likewise, genomic analysis by Huang revealed that *Paenibacillus algicola* (Huang et al., 2022) contains numerous enzymes potentially involved in polysaccharide degradation, such as alginate lyase, agarase, carrageenase. There have been prior reports of agarase from *Photobacterium gaetbulicola* (Jiang et al., 2022) and alginate lyases from *Photobacterium* sp. FC615 (Lu et al., 2019). However, the κ-carrageenase genes of *Photobacterium rosenbergii* has never been documented before, demonstrating the originality and special potential of this strain. It’s worth noting that the presence of many CAZyme genes, including the gene encoding the GH16 family κ-carrageenase illustrates the genetic and evolutionary capacity of this strain to efficiently catabolize carrageenan. This genome not only provides important information for understanding the polysaccharide degradation mechanism of this strain, but also offers new possibilities for the development and discovery of innovative carbohydrate-degrading tool enzymes.

The direct preparation of oligosaccharides by microorganisms is a sustainable alternative to the traditional chemical degradation of strong acids (Zheng L. X. et al., 2023). Compared with traditional chemical approaches, obtaining carrageenan oligosaccharides by direct degradation with GDSX-4 is more environmentally friendly and efficient, reducing the need for harsh reaction conditions and complex purification processes. This has also been demonstrated in some recent similar studies, where marine bacteria have been directly utilized to degrade carob gum in order to obtain its oligomers. Li recently showed that the marine bacterium *Shewanella* sp. LE8 (Li et al., 2024) exhibited better degradation of carrageenan, producing tetrasaccharides from *ι*-carrageenan fermented for 72 h, while *λ*-carrageenan fermented to produce hexasaccharides and tetrasaccharides. Similarly, our isolated the strain GDSX-4 demonstrated its ability to degrade κ-carrageenan into oligosaccharides with well-defined degrees of polymerization. This research presents the strain GDSX-4 as a promising microbial candidate for κ-carrageenase production and establishes a foundation for the swift and efficient preparation of κ-carrageenan oligosaccharides.

In conclusion, a strain of *Photobacterium rosenbergii* GDSX-4 was isolated from the red alga *Gracilaria coronopifolia*, has been subjected to whole genome sequencing, revealing its genomic characteristics and functional gene profile. The whole genome sequencing and analysis of strain GDSX-4 is of great significance for the excavation of novel polysaccharide hydrolase enzymes, and also provides a solid foundation for the future research on the efficient expression and application of κ-carrageenase. The strain GDSX-4 demonstrated significant degradation ability toward carrageenan, sodium alginate, and agar, with the most obvious hydrolysis effect on κ-carrageenan. Under optimal conditions of 40°C and pH 7.0, GDSX-4 was capable of completely hydrolyzing κ-carrageenan to yield κ-neocarabinose, κ-neocarratetraose, and κ-neocarrahexaose within a 48 h period. This indicates that GDSX-4 as a promising microbial candidate for the industrial preparation of κ-carrageenan oligosaccharides.

## Data Availability

The original contributions presented in the study are publicly available. This data can be found here: https://www.ncbi.nlm.nih.gov/, BioProject accession PRJNA1208939.

## Footnotes

^1^https://github.com/marbl/canu/, version 2.0.

^2^ http://topaz.gatech.edu/GeneMark/

^3^ http://www.cazy.org/
